# Light chain–positive rod‐like inclusions in bone marrow plasma cells associated with monoclonal gammopathy of renal significance

**DOI:** 10.1002/ccr3.3362

**Published:** 2020-09-21

**Authors:** Sayoko Okawara, Kenichiro Bando, Junichi Tsukada

**Affiliations:** ^1^ Hematology University of Occupational and Environmental Health Kitakyushu Japan; ^2^ The Second Department of Internal Medicine University of Occupational and Environmental Health Kitakyushu Japan

**Keywords:** monoclonal gammopathy of renal significance, rod‐like inclusion and plasma cell

## Abstract

Rod‐like inclusions have rarely been described in cases of monoclonal gammopathy of renal significance or multiple myeloma. The key finding in our case is the strong κ‐light chain positivity of rod‐like inclusions in plasma cells, which confirms their immunoglobulin nature. Therapeutic benefit of bortezomib was also observed.

## Graphical abstract

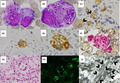

A 68‐year‐old woman was referred to us for κ‐type Bence‐Jones proteinuria (0.3 g/d) and renal insufficiency (serum creatinine 1.77 mg/dL). No serum monoclonal protein was detected in immunofixation electrophoresis. The κ/λ ratio in serum‐free light chain (FLC) was 64.33. Laboratory tests revealed hypophosphatemia (2.4 mg/dL), hypouricemia (1.5 mg/dL), glucosuria, uricosuria, and metabolic acidosis. No bone lytic lesions were observed. Bone marrow aspiration showed 4% of atypical plasma cells with rod‐shaped inclusions in the cytoplasm on Wright‐Giemsa staining (Panels A and B). The cells were positive for CD38 and negative for CD19 on flow cytometry. The inclusions were positive for κ‐light chain (Panels C, D, and E) on immunohistochemical staining of bone marrow aspirate clot sections and negative for acid phosphatase (Panel F).

Renal biopsy exhibited κ‐light chain–positive proximal tubular cells (Panels G and H) containing cytoplasmic microcrystals (Panel I; arrowheads). No Congo red–positive deposits were observed. A diagnosis of light chain proximal tubulopathy with Fanconi syndrome was made. The inclusions disappeared and FLC ratio improved to 2.66 after bortezomib treatment (Figure 1).

**FIGURE 1:**
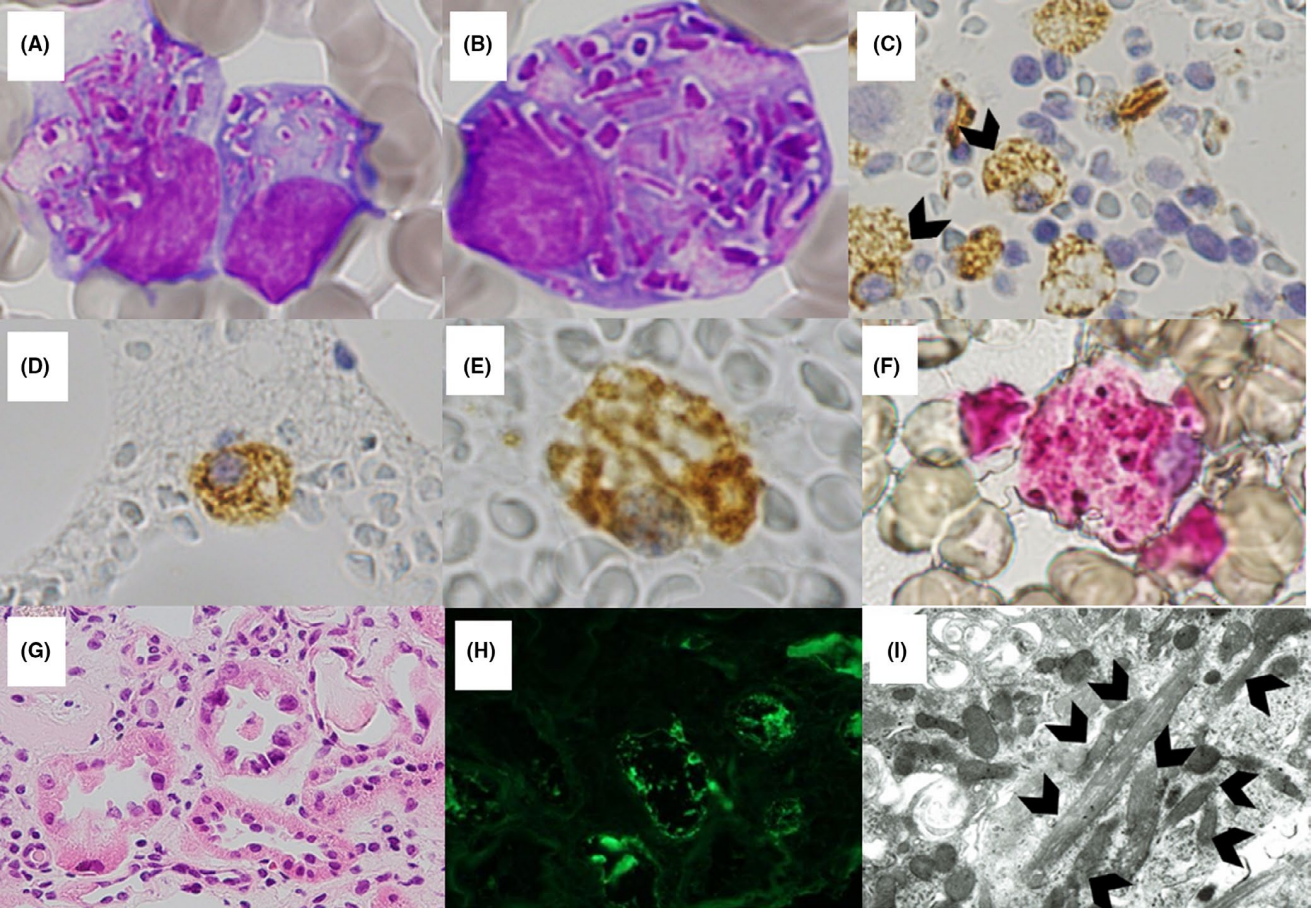
Bone marrow smear showing plasma cells with rod‐shaped inclusions in the cytoplasm (Panels A and B, Wright‐Giemsa staining, original magnification ×1000). Rod‐shaped inclusions in bone marrow plasma cells positively labeled by κ‐light chain on immunohistochemical staining of bone marrow aspirate clot sections (Panels C and D, original magnification ×200; panel E, original magnification ×400). Bone marrow aspirate clot technique is useful in immunohistochemical diagnosis of hematological diseases. Bone marrow aspirate clot was fixed in formalin solution, embedded in paraffin, and sectioned with the standard procedures for bone marrow biopsy, except of decalcification. The same results were obtained from bone marrow aspirate repeated later. No rod‐shaped accumulation acid phosphatase in bone marrow plasma cells (Panel F, original magnification ×400). Renal biopsy showing hypertrophied proximal tubular cells with eosinophilic cytoplasm (Panel G, hematoxylin and eosin staining, original magnification ×200). Positive staining for κ‐light chain in the proximal tubular cytoplasm on immunofluorescence staining (Panel H, original magnification ×200). Proximal tubular cells containing cytoplasmic microcrystals (arrowheads) in electron microscopy (Panel I, original magnification ×6000)

Rod‐like inclusions have been shown to consist of lysosomal enzymes.^1^ However, rod‐like inclusions originating from immunoglobulins have been also reported.^2^ Our findings demonstrated that rod‐like inclusions result from accumulation of κ‐light chain and showed therapeutic benefit of bortezomib treatment.

## CONFLICT OF INTEREST

The authors have no conflicts of interest to declare. This study received no funding from public, private, or not‐for‐profit sectors.

## AUTHOR CONTRIBUTIONS

SO: collected and analyzed the data, and wrote the paper. KB: collected and analyzed the data, and contributed to the revised manuscript. JT: conceived or designed the work, collected and analyzed the data, and wrote the paper. All authors have reviewed and approved the manuscript.

## ETHICAL APPROVAL

Written informed consent was obtained from the patient for the publication of this case and accompanying images.
